# Is there a benefit of ICD treatment in patients with persistent severely reduced systolic left ventricular function after TAVI?

**DOI:** 10.1007/s00392-021-01826-x

**Published:** 2021-03-23

**Authors:** Richard J. Nies, Christian Frerker, Matti Adam, Elmar Kuhn, Victor Mauri, Felix S. Nettersheim, Simon Braumann, Thorsten Wahlers, Stephan Baldus, Tobias Schmidt

**Affiliations:** 1 Department of Cardiology, Heart Center, University of Cologne, Kerpener Str. 62, 50937 Cologne, Germany; 2 Department of Cardiothoracic Surgery, Heart Center, University of Cologne, Kerpener Str. 62, 50937 Cologne, Germany

**Keywords:** Reduced left ventricular function, Aortic stenosis, TAVI, ICD

## Abstract

**Background:**

In patients with severe aortic stenosis (AS) undergoing transcatheter aortic valve implantation (TAVI) and heart failure with severely reduced ejection fraction, prediction of postprocedural left ventricular ejection fraction (LVEF) improvement is challenging. Decision-making and timing for implantable cardioverter defibrillator (ICD) treatment are difficult and benefit is still unclear in this patient population.

**Objective:**

Aims of the study were to analyse long-term overall mortality in TAVI-patients with a preprocedural LVEF ≤ 35% regarding LVEF improvement and effect of ICD therapy.

**Methods and results:**

Retrospective analysis of a high-risk TAVI-population suffering from severe AS and heart failure with a LVEF ≤ 35%. Out of 1485 TAVI-patients treated at this center between January 2013 and April 2018, 120 patients revealed a preprocedural LVEF ≤ 35% and had sufficient follow-up. 36.7% (44/120) of the patients suffered from persistent reduced LVEF without a postprocedural increase above 35% within 1 year after TAVI or before death, respectively. Overall mortality was neither significantly reduced by LVEF recovery above 35% (*p* = 0.31) nor by additional ICD treatment in patients with persistent LVEF ≤ 35% (*p* = 0.33).

**Conclusion:**

In high-risk TAVI-patients suffering from heart failure with LVEF ≤ 35%, LVEF improvement to more than 35% did not reduce overall mortality. Patients with postprocedural persistent LVEF reduction did not seem to benefit from ICD treatment. Effects of LVEF improvement and ICD treatment on mortality are masked by the competing risk of death from relevant comorbidities.

**Graphic abstract:**

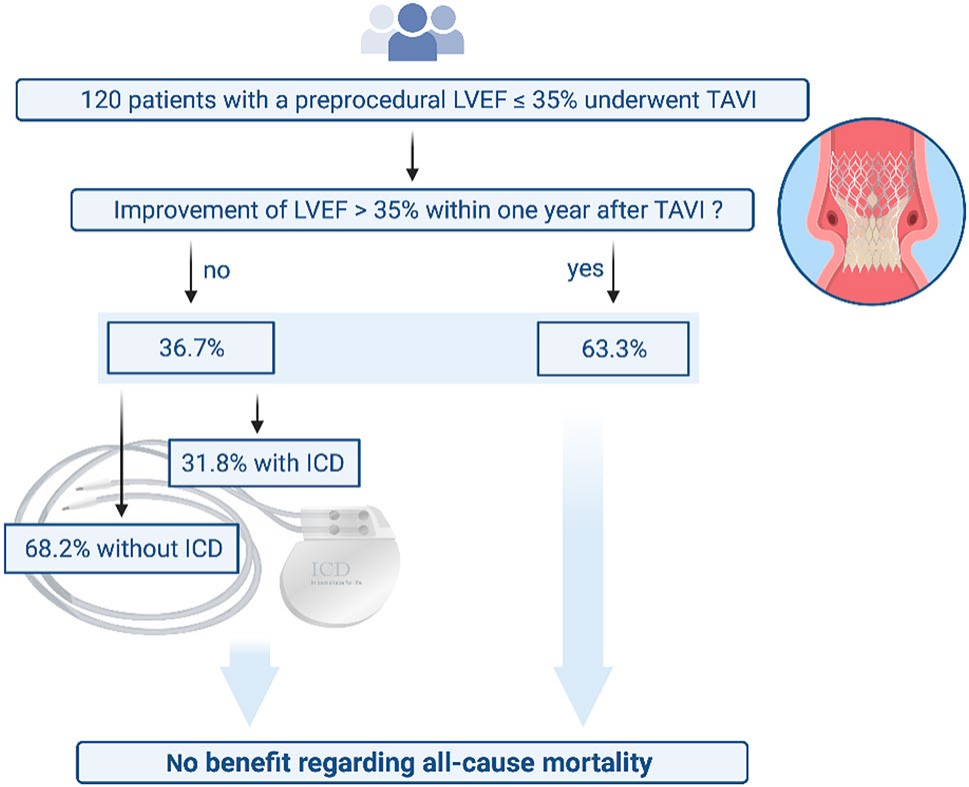

**Supplementary Information:**

The online version contains supplementary material available at 10.1007/s00392-021-01826-x.

## Introduction

Over the last decades, transcatheter aortic valve implantation (TAVI) has become a standard treatment for patients with severe aortic stenosis (AS). Initially, only higher-risk patients seemed to benefit from TAVI, but recently, it has been proven that TAVI is also beneficial in patients at lower risk [1].

Special patient cohorts are patients with reduced systolic left ventricular (LV) function and concomitant AS. Reduced LV-function can result from valvular cause by severe AS, coronary artery disease (CAD) or other factors. In many patients it remains unclear, which factor contributes most to the left ventricular ejection fraction (LVEF) impairment. Patients suffering from heart failure (HF) with reduced ejection fraction (HFrEF) should receive optimal medical treatment (OMT) and concomitant severe CAD, represented by a high Syntax Score, should be treated in conjunction with AS [2, 3]. After TAVI, mid-term mortality of patients with reduced LVEF at baseline is significantly higher compared to patients with a normal LVEF [4].

According to current guidelines, HF-patients with a New York Heart Association (NYHA) class II-III and reduced LVEF (≤ 35%) despite OMT have an indication for an implantable cardioverter defibrillator (ICD) for primary prevention of sudden cardiac death (SCD) [3].

Patients with a history of myocardial infarction (MI) and ischemic cardiomyopathy derive a significant all-cause survival benefit from ICD, whereas studies in patients with non-ischemic cardiomyopathy show controversial results [5–7]. Current guidelines do not differentiate between valvular and other non-ischemic causes of LVEF impairment, although early LVEF improvement after TAVI is seen in up to 62% of these patients [3, 8]. However, individual prediction of LVEF improvement following TAVI remains difficult and optimal choice and timing of cardiac device therapy is still a matter of debate. If additional cardiac resynchronization therapy (CRT) becomes an option after TAVI due to a persistent left bundle branch block (LBBB) or conduction disturbances with the need of right ventricular pacing, decision-making becomes even more complex.

Aims of the study were to evaluate LVEF improvement after TAVI in patients with severe AS and preprocedural LVEF ≤ 35% and to analyse long-term overall mortality regarding postprocedural LVEF improvement and presence of ICD.

## Patients and methods

### Study population

1485 patients underwent TAVI between January 2013 und April 2018 at the Heart Center of the University of Cologne. Inclusion criteria for this study were a preprocedural LVEF of ≤ 35% and a severe AS according to the European Society of Cardiology guideline classification of valvular heart disease [9].

**Fig. 1:**
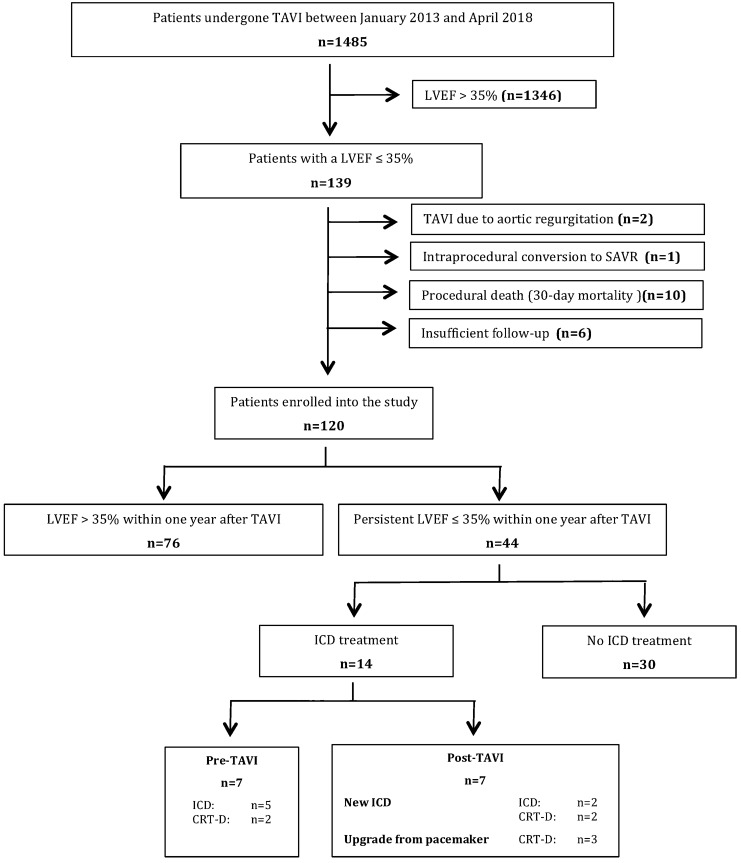
Organizational flowchart of the study

Exclusion criteria were severe aortic regurgitation, intraprocedural conversion to surgical treatment and procedural death defined by 30-day mortality according to the VARC-2 criteria [10]. For follow-up reasons, patients were excluded, if they did not have at least one documented postprocedural transthoracic echocardiography to evaluate systolic LV-function and at least one follow-up at a minimum of 30 days after TAVI. This selection left a final study population of 120 patients (Fig. 1). Preprocedural ICD implantation for primary prevention was withheld in the sense of an individual approach considering possible LVEF recovery after TAVI.

### Transcatheter aortic valve implantation

Choice of intervention [TAVI vs. surgical aortic valve replacement (SAVR)] was made by an institutional Heart-Team consisting of experienced cardiologists, cardiac surgeons and anesthesiologists. Preferred access route for TAVI was transfemoral (82.5%). Balloon-expandable valves were used in 67.5% of the cases, whereas 30.8% of the patients received self-expanding valves. Five of the 120 procedures (4.2%) were "valve-in-valve" procedures.

### Endpoint definitions and follow-up

Clinical and safety endpoints regarding TAVI were defined as recommended by the VARC-2 consensus [10]. Primary endpoint was overall mortality.

Follow-up data were obtained from outpatient care at the Heart Center or in collaboration with resident cardiologists and telephone interviews.

Patients with LVEF increase above 35% were compared with patients, who did not have such a documented LVEF improvement during the follow-up. In addition, comparative analysis of patients with and without absolute LVEF increase ≥ 10% was performed.

Considering clinical status, patients with persistent HFrEF and LVEF below 35% during follow-up received an ICD according to guidelines [3].

### Statistics

Continuous data were presented as mean ± standard deviation (SD). Numbers and percentages were used for categorical data. Univariate subgroup comparisons were performed using unpaired *t* tests for approximately normally distributed variables, Mann–Whitney *U* tests for non-normally distributed variables and Pearson chi-square test or Fisher test for categorical variables. Kolmogorov–Smirnov and Shapiro–Wilk tests were chosen to test for normal distribution. For multivariate analysis, binary multivariate logistic regression with forward and backward selection was used. The Akaike information criterion was calculated to choose the best fitting model. For propensity score matching the variables age, CAD, previous MI, mean aortic pressure gradient (*p*_mean_) < 40 mmHg and baseline LVEF were chosen. Caliper matching with a caliper width of 0.2 was used as matching method [11]. For univariate subgroup comparisons, paired *t* test was used for approximately normally distributed variables and Wilcoxon test was chosen for non-normally distributed variables. Categorical variables were analysed by McNemar test. Kaplan Meier curves were used to visualize differences in overall mortality between subgroups considering ICD implantation date after TAVI. Analyses were limited to 36 months of follow-up. Weighted log-rank and likelihood ratio tests were chosen to compare the results. A *p* value of ≤ 0.05 was considered to be statistically significant. Statistical analysis was performed using R version 4.0.1 (package “Survival Analysis” version 3.2–3) and IBM SPSS Statistics for Macintosh version 25.0.

## Results

### Study population

Mean age of the study population (*n* = 120) was 79.5 ± 6.2 years and 58.3% of the patients were males. 75.8% of the patients suffered from CAD and 31.7% had a history of MI. Mean LVEF was 28.7 ± 5.7% and 32.5% of the patients had a *p*_mean_ ≥ 40 mmHg before TAVI. Mean stroke volume index (SVI) was 29.9 ± 8.4 ml/m^2^. Mean Society of Thoracic Surgery (STS)-Score, Log. Euro-Score and EuroSCORE II were 4.6 ± 3.3, 30.0 ± 17.3 and 10.6 ± 8.8, respectively. 22.5% (27/120) of the study population had a cardiac device before TAVI (15% (18/120) with a permanent pacemaker and 7.5% (9/120) with an ICD).

Stroke rate was 3.3% (4/120) and 5.9% of the patients (7/120) had at least a moderate paravalvular leakage. Mean NYHA class improvement was 0.4 ± 0.9. 27 patients of the study population (*n* = 120) had a cardiac device implantation before TAVI. Patients without pre-existing cardiac device (*n* = 93) received a new cardiac device in 21.5% (20/93) of the cases within the first year after TAVI. 16 patients received a pacemaker [main cause was high-grade atrioventricular block (12/16)] and four patients received an ICD.

### LVEF improvement vs. absence of LVEF improvement after TAVI

Within 1 year after TAVI, 63.3% (76/120) of the study cohort showed an improvement of LVEF to more than 35%, whereas 36.7% (44/120) did not (Fig. 1). Regarding the subgroups with a LVEF above and below 35% during follow-up, mean LVEF was 49.6 ± 7.3% and 27.6 ± 5.3% after 1 year, respectively. Absolute LVEF increase was significantly different between both groups (19.7 ± 9.2% vs. 1.2 ± 6.3%; *p* < 0.001). Patients with LVEF improvement (LVEF > 35% and absolute LVEF increase ≥ 10%) showed a significantly higher NYHA class improvement compared to those with persistent LVEF reduction (*p* = 0.005 and *p* = 0.017).

In a univariate analysis, patients without LVEF recovery had a significantly higher log. Euro-Score as well as EuroSCORE II and showed a higher portion of male gender, pre-existing cardiac devices, previous MI and previous cardiac surgery. Moreover, these patients had a significantly lower *p*_mean_ and LVEF at baseline (Table 1a). Regarding multivariate analysis, previous cardiac surgery, history of MI, male gender and lower initial LVEF were identified as independent predictors for persistent LVEF reduction after TAVI. In patients without LVEF, improvement above 35% solely EuroSCORE II was significantly higher in the matched study collective (Online Resource 1). Patients with and without absolute LVEF increase ≥ 10% showed no significant differences in the presence of CAD or previous MI. Independent predictors for an absolute LVEF increase < 10% were previous cardiac surgery, a lower p_mean_ at baseline and male gender (Online Resource 2).

**Table 1:** (a) Baseline characteristics of subgroups regarding LVEF within 1 year after TAVI; (b) baseline characteristics of patients with persistent LVEF reduction within 1 year after TAVI regarding ICD presence

	a			b		
	Study collective (*n* = 120)			LVEF ≤ 35% after TAVI (*n* = 44)		
	LVEF > 35 after TAVI (*n* = 76)	LVEF ≤ 35% after TAVI (*n* = 44)	*p*	ICD (*n* = 14)	No ICD (*n* = 30)	*p*
Male patients; %	50.0	72.7	0.021	78.6	70.0	0.722
Age (years); mean ± SD	79.9 ± 5.5	79.0 ± 7.4	0.733	76.5 ± 4.9	80.1 ± 8.1	0.022
BMI (kg/m^2^); mean ± SD	26.0 ± 5.2	25.8 ± 4.0	0.742	28.0 ± 4.4	24.7 ± 3.4	0.028
NYHA III/IV; %	88.2	90.9	0.766	85.7	93.3	0.581
CAD; %	72.4	81.8	0.276	71.4	86.7	0.242
Previous myocardial infarction; %	25.0	43.2	0.044*	42.9	43.3	1.000
Previous cardiac surgery; % CABG only SAVR only CABG and SAVR CABG and other valve replacement Others	10.5 6.6 0.0 0.0 1.3 2.6	50.0 34.1 4.5 2.3 0.0 9.1	< 0.001*	42.9 14.3 7.1 0.0 0.0 21.4	53.3 43.3 3.3 3.3 0.0 3.3	0.747
Arterial hypertension; %	85.5	93.2	0.251	92.9	93.3	1.000
Diabetes mellitus; %	43.4	45.5	0.851	21.4	56.7	0.050
Chronic obstructive pulmonary disease; %	19.7	15.9	0.807	7.1	20.0	0.401
Atrial fibrillation; %	48.7	61.4	0.191	57.1	63.3	0.748
Peripheral artery disease; %	21.1	29.5	0.377	14.3	36.7	0.170
GFR; % ≥ 60 ml/min < 60 ml/min dialysis	39.5 57.9 2.6	34.1 65.9 0.0	0.434	35.7 64.3 0.0	33.3 66.7 0.0	1.000
STS-Score; mean ± SD	4.5 ± 3.0	4.9 ± 3.8	0.711	3.2 ± 1.6	5.6 ± 4.3	0.025
EuroSCORE II; mean ± SD	8.9 ± 8.8	13.5 ± 8.2	< 0.001	8.7 ± 4.4	15.7 ± 8.7	0.005
Log. Euro-Score; mean ± SD	26.5 ± 16.5	36.0 ± 17.2	0.002	26.2 ± 12.2	40.6 ± 17.5	0.011
LVEF (%); mean ± SD	30.0 ± 5.2	26.4 ± 5.9	0.002*	24.0 ± 6.9	27.6 ± 5.1	0.097
AVA (cm^2^); mean ± SD	0.66 ± 0.18 (*n* = 74)	0.72 ± 0.17 (*n* = 42)	0.071	0.68 ± 0.13	0.74 ± 0.18 (*n* = 28)	0.398
p_mean_ (mmHg); mean ± SD	36.1 ± 12.0 (*n* = 73)	29.4 ± 13.9 (*n* = 41)	0.008	28.5 ± 13.7 (*n* = 13)	29.8 ± 14.2 (*n* = 28)	0.799
*p*_mean_ < 40 mmHg; %	57.5 (*n* = 73)	85.4 (*n* = 41)	0.003	84.6 (*n* = 13)	85.7 (*n* = 28)	1.000
SVI (ml/m^2^); mean ± SD	30.3 ± 8.8 (*n* = 67)	29.3 ± 7.8 (*n* = 40)	0.666	26.3 ± 5.5 (*n* = 13)	30.8 ± 8.5 (*n* = 27)	0.091
Preprocedural cardiac device; % Pacing ICD	14.5 11.9 2.6	36.4 20.5 15.9	0.008	57.1 7.1 50.0	26.7 26.7 0.0	< 0.001

*AVA* aortic valve area, *BMI* body mass index, *CABG* coronary artery bypass grafting, *CAD* coronary artery disease, *GFR* glomerular filtration rate, *ICD* implantable cardioverter defibrillator, *LVEF* left ventricular ejection fraction, *NYHA* New York Heart Association, *p*_mean_ mean aortic pressure gradient, *SAVR* surgical aortic valve replacement, *SD* standard deviation, *STS* Society of Thoracic Surgery, *SVI* stroke volume index, *TAVI* transcatheter aortic valve implantation

*Statistically significant in multivariate analysis (*p* = 0.05)

Patients with LVEF recovery above 35% received a new pacemaker during the first year after TAVI in 16.9% (11/65), whereas 17.9% (5/28) did in the group with persistent LVEF reduction (*p* = 1.00).

### ICD in patients with persistent HFrEF after TAVI

Out of 44 patients with persistent LVEF ≤ 35%, 14 patients had an ICD. Seven of these patients already had a pre-existing ICD and another seven patients received an ICD during total follow-up (new ICD within 1 year *n* = 4, upgrade from pacemaker during total follow-up *n* = 3). Accordingly, postprocedural ICD implantation rate was 18.9% (7/37) and mean duration to ICD implantation after TAVI was 231 ± 225 days. Additional CRT was present in 50% of the ICD patients (Fig. 1). 30 of 44 patients with persistent LVEF ≤ 35% were not treated with an ICD. In two cases the patients refused a recommended ICD and in three patients active decision for CRT-P instead of a CRT-D was made. Regarding the remaining 25 patients, information of withholding ICD was unknown.

Patients without ICD were significantly older, had higher surgical risk scores, a lower body mass index (BMI) and more often diabetes compared to ICD recipients (Table 1b). Regarding TAVI procedure itself and TAVI associated complications, no significant differences were found between subgroups.

### Overall mortality

Mean follow-up time was 27.2 ± 17.9 months. Overall 2- and 4-year survival of the study population was 65.2 and 36.0%, respectively. Death occurred in 50% (60/120) of all included patients during follow-up. 25% of these 60 patients died from cardiovascular cause, whereas 36.7% suffered a non-cardiovascular death (e.g. sepsis, malignant disease or other causes). Cause of death remained unclear in 38.3% of these cases.

In univariate analysis moderate to severe paravalvular leakage (*p* = 0.72), stroke rate (*p* = 0.12) and new pacemaker implantation rate (*p* = 0.27) did not differ significantly between deceased and living patients, whereas EuroSCORE II did (12.2 ± 10.6 vs. 9.0 ± 6.3; *p* = 0.045).

**Fig. 2:**
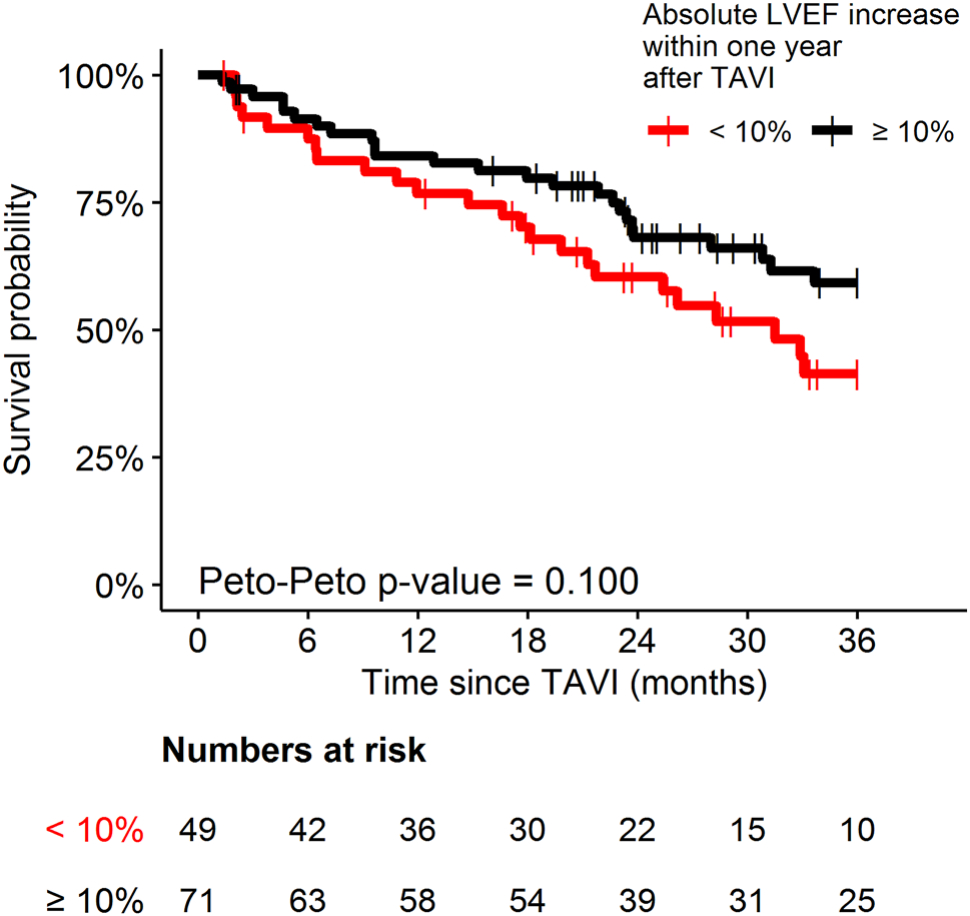
Overall mortality regarding patients with an absolute LVEF increase of 10% or more (black curve) and less than 10% (red curve) within 1 year after TAVI

**Fig. 3:**
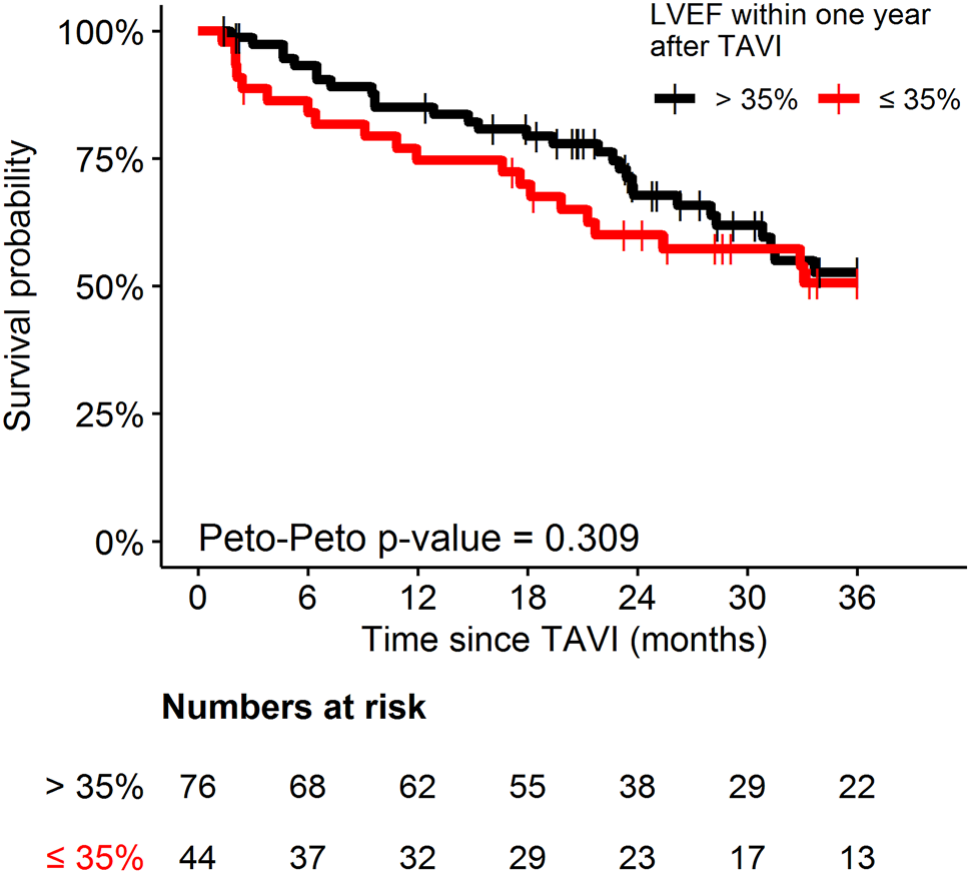
Overall mortality regarding patients with LVEF improvement above 35% (black curve) and patients with persistent LVEF ≤ 35% (red curve) within 1 year after TAVI

The extent of absolute postprocedural LVEF increase tends to have an impact on overall mortality, but differences did not reach statistical significance (Fig. 2, Online Resource 3). Hence, LVEF improvement to more than 35% after TAVI did not reduce overall mortality compared to patients with persistent LVEF reduction (Fig. 3, Online Resource 3). Two-year survival in patients with and without LVEF improvement was 68.1 and 60.1%, respectively (*p* = 0.16). Cardiovascular cause of death was low in both groups (20 vs. 32%).

**Fig. 4:**
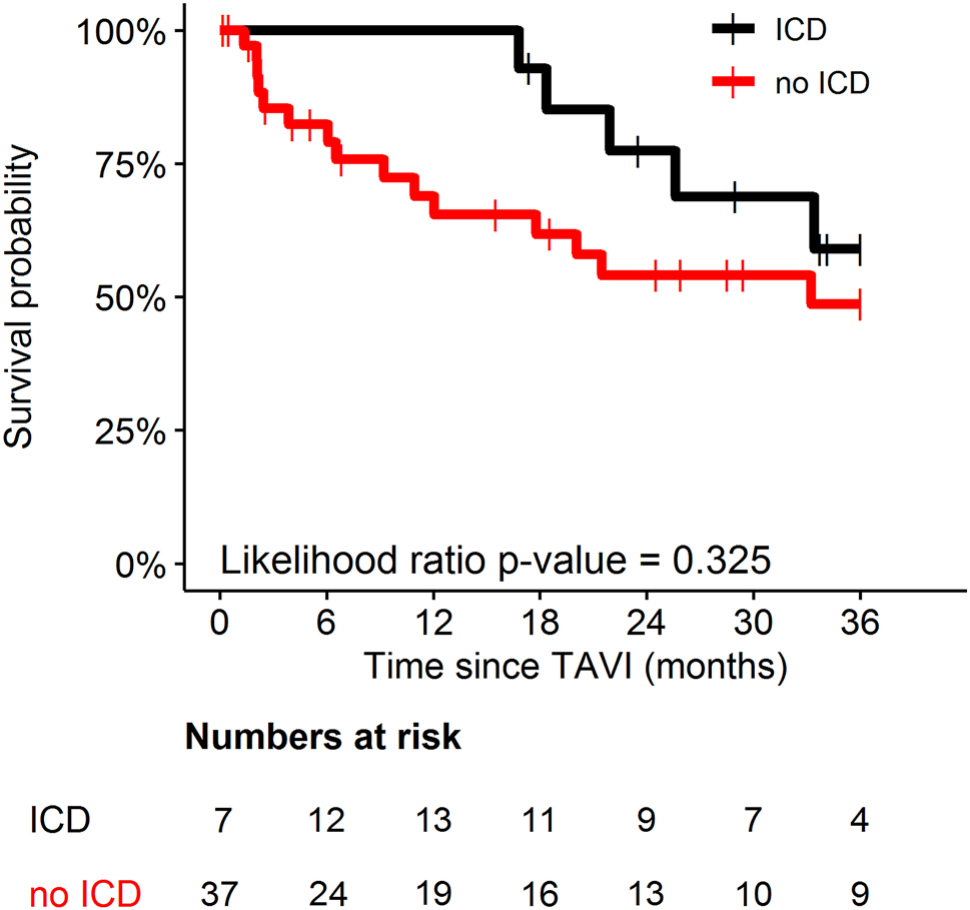
Overall mortality of patients with persistent LVEF ≤ 35% after TAVI regarding presence (black curve) and absence (red curve) of ICD

ICD treatment did not reduce overall mortality in TAVI-patients with persistent LVEF impairment (Fig. 4). Regarding all occurred deaths, proportion of cardiovascular deaths was 31.6% in ICD patients and 33.3% in patients without ICD.

## Discussion

Patients undergoing TAVI with reduced LVEF ≤ 35% and severe AS were analysed. Main findings of this study are:

1. LVEF improvement above 35% and consecutive loss of ICD indication was found in 63% of the patients within 1 year after TAVI.
2. Overall mortality after TAVI was not improved in patients with a LVEF increase above 35% compared to patients with persistent LVEF ≤ 35%.
3. In patients with persistent LVEF reduction ≤ 35% within 1 year after TAVI, ICD did not reduce overall mortality.

### LVEF improvement

Low-flow low-gradient AS due to reduced LVEF is associated with a worse prognosis, but patients benefit from TAVI independent of flow reserve [9, 12, 13]. Still, careful preprocedural diagnostic work-up is of high importance with respect to pseudo-severe AS. However, prediction of postprocedural LVEF increase is difficult because of different entities of cardiomyopathy. Therefore, ICD indication for primary prevention needs to be reevaluated after TAVI and possible LVEF improvement needs to be awaited. Elimination of pressure overload and reverse LV remodelling have shown an LVEF increase above 35% in 63.3% of the study collective. Concordant to other studies, absence of previous MI or cardiac surgery and an initial *p*_mean_ ≥ 40 mmHg have been found to predict LVEF recovery [8, 13, 14]. Although some authors identified lower initial LVEF being predictive for LVEF improvement, the opposite was found in this study [13, 14]. Interestingly, absolute LVEF increase ≥ 10% was independent from CAD and previous MI emphasizing the potential of TAVI when HFrEF is predominately caused by valvular heart disease due to AS. We further believe, that patients without significant LVEF increase after TAVI suffer from an advanced stage of valvular heart disease with extensive myocardial fibrosis (MF)/scar tissue delaying reverse LV remodelling and limiting LVEF increase [15, 16]. Further impact on LVEF recovery is caused by persistent right ventricular pacing or LBBB, which should be avoided because both worsen systolic LV function and outcome after TAVI [17]. Patients with persistent LVEF ≤ 35% showed a significant higher proportion of pre-existing cardiac devices, assuming other factors than AS being responsible for LVEF reduction in some cases.

### Overall mortality

After TAVI 2-year survival rate was 65.2% corresponding to previous observations made in classical low-flow low-gradient AS cohorts [13, 18]. Moderate to severe paravalvular leakage, periprocedural major stroke and new pacemaker implantation did not have significant impact on overall mortality in this study, although these complications are known to be adverse predictors for survival after TAVI [17, 19, 20].

Although absolute LVEF improvement tends to be beneficial, overall mortality in patients with a postprocedural LVEF improvement above 35% was not reduced compared to patients with persistent HFrEF. Of note, even after matching the study collective for CAD and previous MI, patients without LVEF recovery were at higher risk according to EuroSCORE II. On the one hand, the absence of mortality benefit appears counterintuitive, but on the other hand, it underlines, that relevant secondary diagnoses in this typical TAVI-cohort mask the effect of LVEF improvement on overall mortality [21]. This multimorbidity was represented by high surgical risk scores in general. In addition, the rate of cardiovascular death was low in both subgroups.

However, since TAVI becomes an option even in younger lower-risk patients, LVEF increase might have an impact on prognosis, but for an old and multimorbid collective LVEF improvement did not reduce mortality significantly [1].

### Impact of ICD

In this study cohort severe comorbidities were frequently present, competing with the risk of SCD and influencing quality of life [22]. Patients with ischemic HF following MI showed a significant all-cause survival benefit when treated with ICD [5]. Older and specific non-ischemic HF-patients, who are less prone to life-threatening arrhythmias, did not derive such all-cause mortality benefit from ICD in the DANISH trial [6]. Since CRT improves LVEF and survival compared to OMT, the role of additional ICD is a matter of debate in patients with non-ischemic cardiomyopathy [23–25]. Because large CRT-P trials had significantly lower rates of SCD in comparison to HF trials, lacking benefit of ICD in older non-ischemic HF-patients is in doubt [26].

A RCT meta-analysis concluded a beneficial effect of an ICD for primary prevention in patients older than 75 years despite a higher overall mortality risk due comorbidities [22]. Other authors found ICD in elderly being less beneficial [27, 28]. Because perioperative complications and inappropriate shocks are independent from age, age alone should not be the reason to withhold ICD [29]. However, in this study patients without ICD were indeed significantly older and had higher risk scores compared to ICD recipients, showing caution regarding ICD implantation in elderly patients.

Recently, a high extent of MF in TAVI-patients treated for severe AS has been found to be an independent predictor for cardiovascular mortality, predominately arrhythmias [16]. Hence, a causal relationship between pathological LV remodelling, MF and cardiovascular mortality has been hypothesized. This may lead to advanced assessment of MF by additional magnetic resonance imaging to guide ICD treatment in these patients [16].

However, this study describes a highly selective patient population suffering—at least to some extent—from valvular heart disease with the possibility of significant LVEF increase after TAVI. Studies for this type of HF and ICD are missing, but the benefit of an ICD in a multimorbid elderly TAVI collective seems to disappear. Hence, individual risk stratification is needed and ICD implantation should be discussed with caution.

### Study limitations

Several limitations of this single-center, retrospective, observational, non-randomized study are noteworthy. Despite the large overall number of patients treated, the number of patients with HFrEF and larger follow-up is limited, reducing the power of the study. Transthoracic echocardiography has an inter-investigator variation concerning determination of LVEF, however, auto EF mode reduces this limitation to some extent. According to low-flow low-gradient AS dobutamine stress echocardiography was not routinely performed. Of note, using a fixed LVEF cut-off value of 35% to define subgroups, under- or overrepresentation of absolute LVEF improvement might be present in single cases depending on the baseline LVEF. Regarding cardiac devices, information on pacing modes, stimulation rates and adequate or inadequate shock therapy was not reliably detectable. Notably, due to the number of patients, ICD subgroup included patients with preprocedural and postprocedural implanted ICDs.

## Conclusion

In high-risk patients undergoing TAVI for severe AS in the presence of HFrEF with LVEF ≤ 35%, LVEF increase above 35% was likely within 1 year and only 36.7% of the patients suffered from persistent high-grade deterioration of LVEF. In patients with LVEF recovery overall mortality was not reduced compared to patients with persistent LVEF ≤ 35%. Moreover, in patients with persistent LVEF impairment ICD did not reduce overall mortality. Beneficial effects of LVEF increase and ICD treatment are masked by the competing risk of death from comorbidities. Large randomized controlled studies and multicenter studies are necessary to determine the impact of ICD in patients with persistent HFrEF after TAVI.

## Supplementary Information

Below is the link to the electronic supplementary material.

Supplementary file1 (PDF 106 KB)

Supplementary file2 (PDF 114 KB)

Supplementary file3 (PDF 305 KB)
