# Supplementary material for: Is there a benefit of ICD treatment in patients with persistent severely reduced systolic left ventricular function after TAVI?
Source: Clin Res Cardiol. 2021 Mar 23;111(5):492–501. doi: 10.1007/s00392-021-01826-x (PMC9054877; doi:10.1007/s00392-021-01826-x)
Supplement: Supplementary file 1 — Supplementary file1 (PDF 106 KB) [file 392_2021_1826_MOESM1_ESM.pdf]

Is there a benefit of ICD treatment in patients with persistent severely reduced systolic left ventricular function after TAVI?

Clinical Research in Cardiology

- Online Resource 1 -

Richard J. Nies[1] MD, Christian Frerker[1] MD, Matti Adam[1] MD, Elmar Kuhn[2] MD, Victor Mauri[1] MD, Felix S. Nettersheim[1] MD, Simon Braumann[1] MD, Thorsten Wahlers[2] MD, Stephan Baldus[1] MD, Tobias Schmidt[1] MD

[1] Department of Cardiology, Heart Center, University of Cologne, Kerpener Str. 62, D-50937 Cologne, Germany

[2] Department of Cardiothoracic Surgery, Heart Center, University of Cologne, Kerpener Str. 62, D-50937 Cologne, Germany

Corresponding author:

Dr. med. Richard Nies

Department of Cardiology

University of Cologne

Kerpener Straße 62

D-50937 Köln, Germany

Phone: +49 221 47876653

E-mail: richard.nies@uk-koeln.de

Online Resource 1 Baseline characteristics of matched subgroups regarding LVEF > 35% within one year after TAVI

	**Matched study collective (n=62)**		
	**LVEF > 35% after TAVI (n=31)**	**LVEF ≤ 35% after TAVI (n=31)**	**p**
Male patients;%	54.8	80.6	0.039
Age (years);mean±SD	78.9±5.9	79.1±7.2	0.888
BMI (kg/m^2);mean±SD	25.7±5.9	25.8±3.7	0.399
NYHA III/IV;%	90.3	93.5	1.000
CAD;%	83.9	77.4	0.727
Previous myocardial infarction;%	48.4	45.2	1.000
Previous cardiac surgery;%<br>CABG only<br>SAVR only<br>CABG and SAVR<br>CABG and other valve replacement<br>Others	12.9<br>9.7<br>0.0<br>0.0<br>0.0<br>3.2	51.6<br>32.3<br>6.5<br>0.0<br>0.0<br>12.9	0.004
Arterial hypertension;%	87.1	93.5	0.687
Diabetes mellitus;%	38.7	48.4	0.581
Chronic obstructive pulmonary disease;%	25.8	19.4	0.791
Atrial fibrillation;%	45.2	67.7	0.167
Peripheral artery disease;%	32.3	19.4	0.388
GFR; % ≥ 60 ml/min<br>< 60ml/min<br>dialysis	35.5<br>58.1<br>6.5	35.5<br>64.5<br>0.0	n.a.
STS-Score;mean±SD	4.9±2.9	4.2±2.7	0.262
EuroSCORE II;mean±SD	9.3±6.4	12.6±8.8	0.035
Log. Euro-Score;mean±SD	28.9±14.7	34.7±17.7	0.147
LVEF (%);mean±SD	27.9±5.0	27.6±4.8	0.700
AVA (cm^2);mean±SD	0.65±0.16 (n=30)	0.72±0.18 (n=30)	0.203
p_{mean} (mmHg);mean±SD	32.7±8.9	31.4±15.0	0.399
p_{mean} < 40 mmHg;%	74.2	80.6	0.687
SVI (ml/m^2);mean±SD	28.5±8.4 (n=27)	28.8±6.6 (n=27)	0.866
Preprocedural cardiac device;%<br>Pacing<br>ICD	16.1<br>9.7<br>6.5	38.7<br>29.0<br>9.7	0.123

AVA=aortic valve area; BMI=body mass index; CABG=coronary artery bypass grafting; CAD=coronary artery disease; GFR=glomerular filtration rate; ICD=implantable cardioverter defibrillator; LVEF=left ventricular ejection fraction; NYHA=New York Heart Association; p_{mean}=mean aortic pressure gradient SAVR=surgical aortic valve replacement; SD=standard deviation; STS=Society of Thoracic Surgery; SVI=stroke volume index; TAVI=transcatheter aortic valve implantation
