# Supplementary material for: Is there a benefit of ICD treatment in patients with persistent severely reduced systolic left ventricular function after TAVI?
Source: Clin Res Cardiol. 2021 Mar 23;111(5):492–501. doi: 10.1007/s00392-021-01826-x (PMC9054877; doi:10.1007/s00392-021-01826-x)
Supplement: Supplementary file 2 — Supplementary file2 (PDF 114 KB) [file 392_2021_1826_MOESM2_ESM.pdf]

Is there a benefit of ICD treatment in patients with persistent severely reduced systolic left ventricular function after TAVI?

Clinical Research in Cardiology

- Online Resource 2 -

Richard J. Nies[1] MD, Christian Frerker[1] MD, Matti Adam[1] MD, Elmar Kuhn[2] MD, Victor Mauri[1] MD, Felix S. Nettersheim[1] MD, Simon Braumann[1] MD, Thorsten Wahlers[2] MD, Stephan Baldus[1] MD, Tobias Schmidt[1] MD

[1] Department of Cardiology, Heart Center, University of Cologne, Kerpener Str. 62, D-50937 Cologne, Germany

[2] Department of Cardiothoracic Surgery, Heart Center, University of Cologne, Kerpener Str. 62, D-50937 Cologne, Germany

Corresponding author:

Dr. med. Richard Nies

Department of Cardiology

University of Cologne

Kerpener Straße 62

D-50937 Köln, Germany

Phone: +49 221 47876653

E-mail: richard.nies@uk-koeln.de

Online Resource 2 Baseline characteristics of unmatched (a) and matched (b) subgroups regarding absolute LVEF increase ≥ 10% within one year after TAVI

	a				b		
	Study collective (n=120)				Matched study collective (n=78)		
	Absolute LVEF increase ≥ 10% within one year after TAVI (n=71)	Absolute LVEF increase < 10% within one year after TAVI (n=49)	p		Absolute LVEF increase ≥ 10% within one year after TAVI (n=39)	Absolute LVEF increase < 10% within one year after TAVI (n=39)	p
Male patients;%	50.7	69.4	0.059		48.7	74.4	0.052
Age (years);mean±SD	79.4±6.0	79.8±6.6	0.682		79.1±6.2	79.3±6.6	0.729
BMI (kg/m^2);mean±SD	26.0±5.3	25.9±3.9	0.588		25.5±5.6	25.6±4.0	0.512
NYHA III/IV;%	85.9	93.9	0.235		84.6	92.3	0.453
CAD;%	71.8	81.6	0.280		71.8	79.5	0.508
Previous myocardial infarction;%	25.4	40.8	0.110		35.9	33.3	1.000
Previous cardiac surgery;%<br>CABG only<br>SAVR only<br>CABG and SAVR<br>CABG and other valve replacement<br>Others	15.5<br>9.9<br>0.0<br>1.4<br>1.4<br>2.8	38.8<br>26.5<br>4.1<br>0.0<br>0.0<br>8.1	0.005*		17.9<br>12.8<br>0.0<br>0.0<br>2.6<br>2.6	35.9<br>23.1<br>5.1<br>0.0<br>0.0<br>7.7	0.118
Arterial hypertension;%	87.3	89.8	0.778		87.2	87.2	1.000
Diabetes mellitus;%	43.7	44.9	1.000		38.5	43.6	0.824
Chronic obstructive pulmonary disease;%	19.7	16.3	0.811		25.6	20.5	0.774
Atrial fibrillation;%	50.7	57.1	0.577		46.2	61.5	0.263
Peripheral artery disease;%	22.5	26.5	0.667		30.8	17.9	0.267
GFR; % ≥ 60 ml/min<br>< 60ml/min<br>dialysis	40.8<br>56.3<br>2.8	32.7<br>67.3<br>0.0	0.290		46.2<br>51.3<br>2.6	35.9<br>64.1<br>0.0	n.a.
STS-Score;mean±SD	4.6±3.1	4.7±3.6	0.779		5.1±3.5	4.1±2.3	0.258
EuroSCORE II;mean±SD	9.7±9.2	11.9±8.1	0.035		11.0±11.2	10.8±7.0	0.426
Log. Euro-Score;mean±SD	28.4±17.1	32.2±17.7	0.212		31.1±18.0	31.0±16.7	1.000
LVEF (%);mean± SD	28.5±5.9	29.0±5.3	0.644		27.7±5.7	28.8±5.4	0.030
AVA (cm^2);mean±SD	0.66±0.18 (n=69)	0.71±0.16 (n=47)	0.098		0.67±0.19 (n=37)	0.69±0.15 (n=37)	0.507
p_{mean} (mmHg);mean±SD	35.9±12.5 (n=68)	30.4±13.3 (n=46)	0.028		31.9±12.9	31.9±13.6	0.685
p_{mean} < 40 mmHg;%	57.4	82.6 (n=47)	0.008		76.9	79.5	1.000
SVI (ml/m^2);mean±SD	30.1±9.0 (n=63)	29.8±7.5 (n=44)	0.950		28.8±8.2 (n=36)	28.7±6.2 (n=36)	0.514
Preprocedural cardiac device;%<br>Pacing<br>ICD	15.5<br>11.3<br>4.2	32.6<br>20.4<br>12.2	0.074		20.5<br>12.8<br>7.7	33.4<br>23.1<br>10.3	0.478

*=statistically significant in multivariate analysis (p=0.05); AVA=aortic valve area; BMI=body mass index; CABG=coronary artery bypass grafting; CAD=coronary artery disease; GFR=glomerular filtration rate; ICD=implantable cardioverter defibrillator; LVEF=left ventricular ejection fraction; NYHA=New York Heart Association; p_{mean}=mean aortic pressure gradient; SAVR=surgical aortic valve replacement; SD=standard deviation; STS=Society of Thoracic Surgery; SVI=stroke volume index; TAVI=transcatheter aortic valve implantation
