# Supplementary material for: Is there a benefit of ICD treatment in patients with persistent severely reduced systolic left ventricular function after TAVI?
Source: Clin Res Cardiol. 2021 Mar 23;111(5):492–501. doi: 10.1007/s00392-021-01826-x (PMC9054877; doi:10.1007/s00392-021-01826-x)
Supplement: Supplementary file 3 — Supplementary file3 (PDF 305 KB) [file 392_2021_1826_MOESM3_ESM.pdf]

Is there a benefit of ICD treatment in patients with persistent severely reduced systolic left ventricular function after TAVI?

Clinical Research in Cardiology

- Online Resource 3 -

Richard J. Nies[1] MD, Christian Frerker[1] MD, Matti Adam[1] MD, Elmar Kuhn[2] MD, Victor Mauri[1] MD, Felix S. Nettersheim[1] MD, Simon Braumann[1] MD, Thorsten Wahlers[2] MD, Stephan Baldus[1] MD, Tobias Schmidt[1] MD

[1] Department of Cardiology, Heart Center, University of Cologne, Kerpener Str. 62, D-50937 Cologne, Germany

[2] Department of Cardiothoracic Surgery, Heart Center, University of Cologne, Kerpener Str. 62, D-50937 Cologne, Germany

Corresponding author:

Dr. med. Richard Nies

Department of Cardiology

University of Cologne

Kerpener Straße 62

D-50937 Köln, Germany

Phone: +49 221 47876653

E-mail: richard.nies@uk-koeln.de

Online Resource 3a

Overall mortality of matched subgroups with an absolute LVEF increase of 10% or more (black curve) and less than 10% (red curve) within one year after TAVI

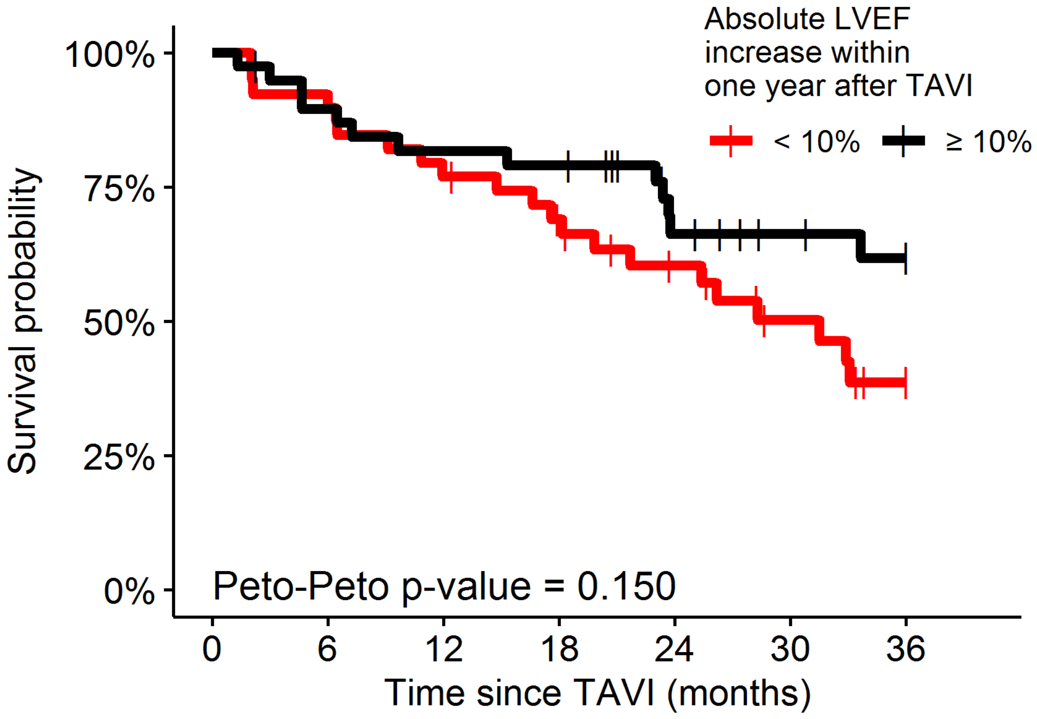

Numbers at risk (matched cohort)

< 10%	39	36	30	25	19	13	8
≥ 10%	39	34	31	30	20	16	14

Online Resource 3b

Overall mortality of matched subgroups with LVEF improvement above 35% (black curve) and patients with persistent LVEF ≤ 35% (red curve) within one year after TAVI

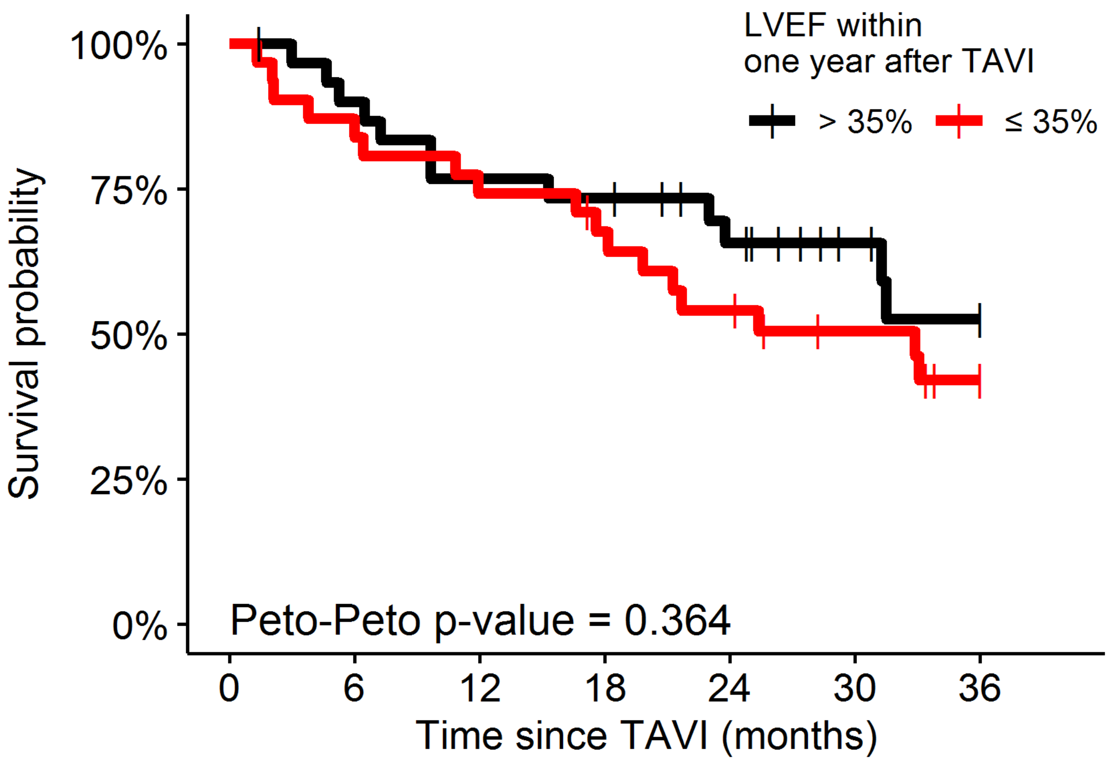

Numbers at risk (matched cohort)

> 35%	31	27	23	22	17	11	8
≤ 35%	31	27	23	20	16	12	8
